# *Casas Maternas* in the Rural Highlands of Guatemala: A Mixed-Methods Case Study of the Introduction and Utilization of Birthing Facilities by an Indigenous Population

**DOI:** 10.9745/GHSP-D-15-00266

**Published:** 2016-03-25

**Authors:** Ira Stollak, Mario Valdez, Karin Rivas, Henry Perry

**Affiliations:** a Curamericas Global, Raleigh, NC, USA; b Curamericas/Guatemala, Calhuitz, Guatemala; c Centro Universitario de Occidente, Quetzaltenango, Guatemala; d Johns Hopkins Bloomberg School of Public Health, Baltimore, MD, USA

## Abstract

**Background:**

An international NGO, with financial and managerial support from “partner” communities, established *Casas Maternas* (birthing facilities) in 3 municipalities in the isolated northwestern highlands of the department of Huehuetenango in Guatemala—an area with high maternal mortality ratio (338 maternal deaths per 100,000 live births). Traditional birth attendants are encouraged to bring patients for delivery at the *Casas Maternas*, where trained staff are present and access to referral care is facilitated.

**Methods:**

We conducted a mixed-methods study in San Sebastian Coatán municipality to assess the contribution of 2 *Casas Maternas* to health facility deliveries among partner and non-partner communities, with particular emphasis on equity in access. We surveyed all women who delivered in the study area between April 2013 and March 2014, the first full year in which both *Casas Maternas* in the study area were operating. In addition, using purposive sampling, we conducted in-depth interviews with 22 women who delivered and 6 focus group discussions with 42 community leaders, traditional birth attendants, and *Casas Maternas* staff members. We analyzed the quantitative data using descriptive statstics and the qualitative data with descriptive content analysis.

**Results:**

Of the 321 women eligible for inclusion in the study, we surveyed 275 women (14.3% could not be located or refused to participate). Between April 2013 and March 2014, 70% of women living in partner communities delivered in a health facility (54% in a *Casa Materna*) compared with 30% of women living in non-partner communities (17% in a *Casa Materna*). There was no statistically significant difference in uptake of the *Casa Materna* by maternal education and only a weak effect by household wealth. In contrast, distance from the *Casa Materna* had a pronounced effect. Traditional birth attendants were strong advocates for utilization of the *Casa Materna* and played an important role in the decision regarding where the birth would take place. In addition, the program’s outreach component, in which peer volunteers visit homes to promote healthy behaviors and appropriate use of health facilities, was identified as a key factor in encouraging mothers to deliver in facilities.

**Discussion:**

The *Casa Materna* approach to strengthening maternity care as developed by Curamericas has potential to increase health facility utilization in isolated mountainous areas inhabited by an indigenous population where access to government services is limited and where maternal mortality is high. The approach shows promise for broader application in Guatemala and beyond.

## Abstract (precis)

In an isolated mountainous area of Guatemala with high maternal mortality, an NGO-sponsored approach engaged communities to operate local, culturally appropriate birthing facilities and is achieving high and equitable utilization. Likely success factors:

- **Community engagement and ownership**
- **Close location of facilities**
- **Perceived high quality of services**
- **Engagement of traditional birth attendants in the birthing process and as advocates for facility use**

## INTRODUCTION

Although Guatemala has made strong national progress in reducing its maternal mortality ratio (MMR) to 88 maternal deaths per 100,000 live births,^1^ this national average hides marked regional and ethnic disparities. The MMR for indigenous women (163 per 100,000) is twice that of non-indigenous women (78 per 100,000), and indigenous women account for 71% of the country’s maternal deaths compared with 54% of the country’s births.^2^ Furthermore, the national percentage of deliveries that take place in facilities is 29% for indigenous women and 70% for non-indigenous women.^2^

Guatemala’s National Study of Maternal Mortality describes the problem of disparities in maternal health outcomes as follows^2^:

The departments most affected by maternal mortality are those that have the highest levels of poverty, rurality, and indigenous population, principally Mayan, as well as the lowest levels of education and highest levels of fertility … This convergence of factors is no coincidence but … is caused by inequitable access to health and human development resources, which is rooted in a history of exclusion and social, economic, and ethnic discrimination that persists.

Key factors accounting for these disparities are culture and language, geographic barriers to accessing services, shortage of rural health personnel, and poverty. Mayan women are less inclined to use Ministry of Health (MOH) facilities because the staff members rarely speak their language, and their cultural practices are prohibited and even scorned. They complain of poor treatment by the Ladino/mestizo (non-indigenous) staff and cite this as a reason for not using the facilities. The MOH facilities are too far away, and dangerous mountainous roads often must be traversed in order to reach them. There is no local or community accountability of the MOH services. There are frequent shortages of staff, and services are frequently unavailable. Furthermore, there is high turnover of MOH staff members who are from a non-indigenous culture and are eager to relocate to an area closer to their home of origin and families.

Guatemala’s pronounced ethnic disparities in maternal health are a result of cultural, language, and geographic barriers to accessing services as well as a shortage of rural health personnel.

The government has made various efforts to address this problem including: (1) “indigenizing” its health staff serving Mayan populations and making services more culturally acceptable, (2) developing a cadre of women authorized to verify and report on the quality of maternity services provided in indigenous areas, and (3) providing training and support to traditional birth attendants (known as *comadronas*) along with formal registration with the government. Special projects have also been implemented over the years, including MotherCare’s government-managed *Casas Maternas*^3^ in the 1990s and Project Concern’s *Casas Maternas* around Lake Atitlan.^4^^,^^5^ These *Casas Maternas* are comprehensive centers for managing high-risk pregnancies referred from outlying areas. Limited in scope, these approaches have not been able to comprehensively address the more complex issue of establishing “community-friendly” facilities for routine delivery with high-quality, nearby, around-the-clock medical care for mothers and newborns and support during childbirth from *comadronas*.

Guatemala’s Northwest Region and the department of Huehuetenango, which are predominantly Mayan, have among the highest maternal mortality ratios (MMRs) in Guatemala (226 per 100,000 live births).^6^ Through its program of routine systematic home visitation and registration of vital events, Curamericas documented over a 10-year period in the municipalities covered by its programs 104 maternal deaths among 30,780 births.^7^ This represents an MMR of 338 per 100,000 live births, one of the highest reported in the Western hemisphere.

**FIGURE 1.**
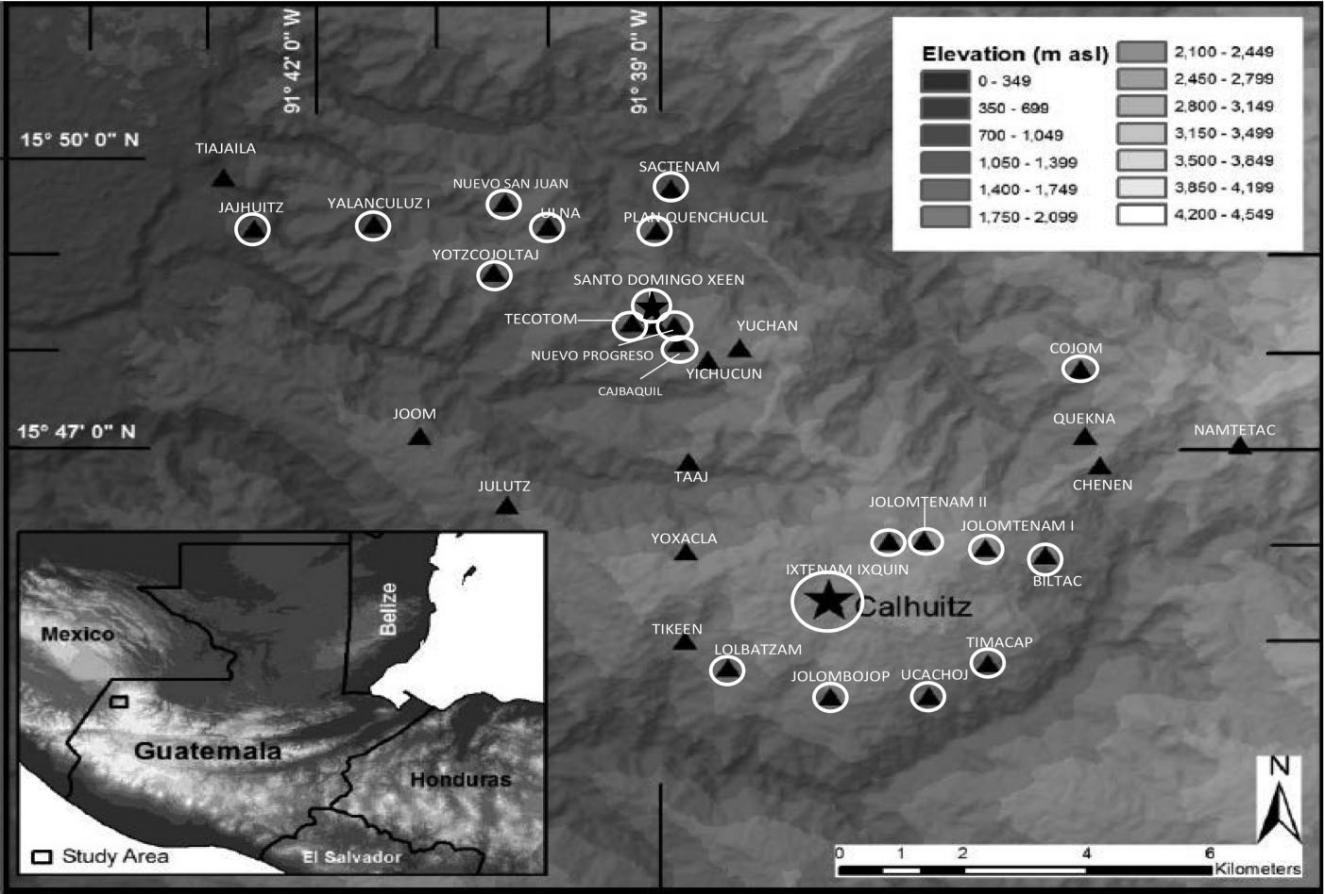
Location of Study Communities in the San Sabastian Coatán Municipality of the Northwestern Highlands of Guatemala ⍟ Partner communities with a *Casa Materna* in operation (Calhuitz and Santo Domingo). ▴ Partner communities in close geographic proximity to a *Casa Materna*. ▴ Non-partner communities.

To address these disparities, Curamericas/Guatemala, in collaboration with Curamericas Global (an international NGO), began a project in 2011 to expand equitable access to and use of maternal and newborn health services. The project worked in a catchment area comprising 28,000 women of reproductive age in 3 isolated municipalities in the department of Huehuetenango (Figure 1).

Curamericas had been working in this isolated part of the department of Huehuetenango since 2002 to reduce the mortality of mothers and children through community-based primary health care services and community engagement. A 2011 survey in the area revealed that 89% of deliveries were still occurring in the homes and 81% were attended by *comadronas*.^7^ The extreme geographic isolation of the communities, lack of transport, and cultural traditions contributed to this situation.

Beginning in 2011, Curamericas built on its engagement in the 3 municipalities where it had been working previously to initiate activities in 89 communities with a combined population of 42,755 (Phase 1). In Phase 2, which began in 2013, the project extended its activities to 94 additional communities with a combined population of 54,867. The project integrates 3 key methodologies:

- A census-based, impact-oriented (CBIO) methodology to mobilize communities and ensure equitable coverage of services^8^
- A Care Group methodology,^9^ which uses volunteer female community peer educators to motivate behavior change and generate demand for maternal/newborn services
- The *Casas Maternas* of Curamericas/Guatemala,^10^ which provide local access to community-based, culturally appropriate maternal services for routine deliveries, in contrast to MotherCare’s and Project Concern’s *Casas Maternas* model, described above, which focus on high-risk pregnancies

In brief, after working in partnership with communities to identify all households, the project recruited 1 woman volunteer for every 10–12 households with a mother and young children to serve as a volunteer peer educator. The volunteers shared key maternal and child health messages with the mothers every 2 weeks through home visits or during meetings with a few neighbors. A group of 10–12 volunteer peer educators met every 2 weeks as a Care Group with a paid facilitator to learn a new message to share with their neighbors, to discuss their activities during the previous 2 weeks, and to report any new vital events.

From the outset of its work in 2002, Curamericas has developed a relationship of respect and collaboration with *comadronas*. Being careful not to interfere in the relationship between families and *comadronas*, Curamericas recognized that *comadronas* are inextricably embedded in the local culture and are thus essential partners for improving maternal and newborn care. Since the work of Curamericas extends down to each household, the program has been able to develop and maintain contact with the *comadronas.* The Box provides further details about the program.

**BOX. What is a *Casa Materna*?**

*Casas Maternas* provide an alternative response to the challenges that Ministry of Health (MOH) services present. These maternity centers provide: (1) scientifically validated medical care with cultural and linguistic adaptation; (2) physical proximity to the patient’s home; (3) community ownership and accountability; and (4) use of lower-level health staff who are willing to live in the area and are usually from a local community. In contrast to MOH facilities generally, at all the *Casas Maternas* the staff members speak the local Mayan dialect, and traditional cultural practices—including the presence of family—are respected.

*Casas Maternas*, as developed and implemented by Curamericas in collaboration with local communities, consist of a simple physical facility, a small number of trained staff, some basic supplies and equipment, and a management and financial support structure embedded in the community. At the time of writing of this article, in the Curamericas program area of almost 100,000 people, there were 4 operating *Casas Maternas*.

**Community Engagement**

Curamericas engages with communities when planning and operating *Casas Maternas*, responding to their interests and needs. In all cases, there is a “micro-region” of communities formed that want to support the development and management of the *Casa Materna* because of their high maternal mortality and distance from MOH facilities. All communities sign a formal agreement. A committee of community members works with the Curamericas program leadership. The community provides the land and labor to construct the facility, while Curamericas provides the materials and the equipment needed to operate it. The managing committee takes responsibility for providing the *Casa Materna* with food and for keeping the facility clean and maintained. The committee also manages emergency referral transport and the transport insurance program (described further below).

**The Physical Infrastructure**

Each *Casa Materna* has a reception area, a traditional Mayan kitchen with a wood-burning stove, potable filtered drinking water, and a bathroom with indoor plumbing (including a toilet, sink, and shower). There is an examination room, a birthing room, a recovery/postpartum room with 2 beds, and an adjacent *chuj* (traditional sweat lodge). There is also a small pharmacy. Outside, there is a patio with a faucet for washing dishes and clothes. The mother and her family can use the kitchen to prepare traditional meals, and the patio to wash and clean dishes.

**The Staff and Their Capabilities**

Each *Casa Materna* is staffed by an auxiliary nurse and 2 support women. The auxiliary nurse performs most deliveries, but the support women are trained to do deliveries in the auxiliary nurse’s absence. The auxiliary nurse is Mayan, from the local municipality, and speaks the local Mayan dialect. Auxiliary nurses receive a 12-month course from the MOH. After then being hired by Curamericas, they receive a 2-month internship at the Calhuitz *Casa Materna*, where a highly experienced obstetric nurse supervisor is stationed along with a readily available physician with training in obstetrics. In addition, there are 2 obstetric registered nurses (RNs), who serve as teachers and supervisors for 2 *Casas Maternas*.

The deliveries occur in a clean and safe environment; there is a dedicated delivery room in the *Casa Materna*, where the staff use the 3 “essential newborn actions” (clean cord care, thermal care, and immediate breastfeeding with colostrum) and active management of the third stage of labor (AMTSL), including routine administration of oxytocin postpartum, controlled umbilical cord traction, and uterine massage. The staff members are also skilled in “home-based life saving skills” (HBLSS), which includes management of retained placenta (and prompt referral if not effective), emergency care of hemorrhage, and resuscitation of the newborn if needed.

This training is provided by the Curamericas nurse supervisor, an obstetric RN (who provides regular monthly trainings to the *Casa Materna* staff members as well as continuous quality control monitoring of their work using a *Casa Materna* staff training guide). She and the *Casa Materna* staff have been trained in HBLSS by nurse-midwives from the American College of Nurse Midwives. The staff received this training in the summer of 2012 and again in the fall of 2013. The staff is also trained in behavior change communication (BCC) concerning antenatal care, the importance of health facility births, postpartum care, and recognition of and prompt response to danger signs during pregnancy, delivery, and the postpartum period, as well as the importance of exclusive breastfeeding and how to do it. They deliver these lessons to support groups of pregnant and lactating women that meet at the *Casa Materna*. They are also trained by the nurse supervisor to provide family planning, carry out antenatal and postpartum care checks, perform Papanicolaou tests to screen for uterine cancer, and provide maternal nutritional counseling.

The auxiliary nurses have taken the American College of Nurse Midwives course in safe delivery. They perform AMTSL by using a partogram, uterine massage, controlled umbilical cord traction, and intramuscular injection of oxytocin. They have available for their use intravenous fluids, blood pressure cuffs, thermometers, antibiotics, and obstetrical tools for clamping the umbilical cord and for repairing perineal tears.

**Delivery Care and Referral**

During the period of labor and delivery, the mothers are allowed to assume whatever position is most comfortable for them. Many like to lean against a large plastic ball about 3 feet in diameter. After the delivery, women stay at the *Casa Materna* for a minimum of 8 hours prior to discharge.

In most cases, a *comadrona* selected by the mother is also present and participates in the care of the mother during labor and delivery and afterwards. Local *comadronas* have been integrated into the *Casa Materna* operations—they look after pregnant women for their usual fee, but encourage women to deliver in the *Casa Materna* and accompany them there, where they assist in the delivery. They are trained by *Casa Materna* staff to detect danger signs during pregnancy and the postpartum period and to promptly refer women with complications to the *Casa Materna* if they are delivering at home.

Each *Casa Materna* is equipped with a satellite telephone to communicate if needed with a volunteer ambulance crew based 1–2 hours away (depending on the *Casa Materna*) at a town at the foot of the mountain range. Each *Casa Materna* has drivers and vehicles on call to take a patient in need of referral to meet the ambulance at the foot of the mountain. Transport to the hospital in the city of Huehuetenango (the only referral facility available) for patients costs US$125 to $150, a very large sum of money for the families living in the area. An insurance scheme has been developed by the communities with facilitation by Curamericas, in which families can pay into for about $13. If transport is needed, the insurance covers half the transportation cost.

During the 12-month study period, the 2 *Casas Maternas* had 118 deliveries and 19 referrals to the hospital in Huehuetenango (16.1% of deliveries were referred). The reasons for referral of these 19 patients were: hemorrhage (5, including 3 with placenta previa); prolonged labor (4); transverse lie (4); premature rupture of membranes and premature labor (3); pre-eclampsia (2, including 1 with associated hemorrhage); and other complicating medical problems (1). All patients who were referred accepted the referral. Of these referrals, there were no maternal deaths, 3 stillbirths, and 1 death of a newborn while the mother and newborn were still at the hospital. Thirteen of the 19 referred women underwent cesarean delivery, 4 delivered vaginally, and 2 underwent dilation and curettage (D&C). The cesarean delivery rate among the women coming to the *Casa Materna* during the 12-month study period was 11.0% (13/118). Complications managed at the *Casa Materna* without the need for referral were 6 women with completed abortions (treated with rehydration and antibiotics) and 7 women with footling presentations.

**Availability of Services and Cost to Patients**

Services at the *Casa Materna* are free and available to any woman, whether or not she comes from a partnering community in the “micro-region.” In practice, however, women prefer not to go very far from their home to deliver. The population of the partner communities in the “micro-regions” that is responsible for a *Casa Materna* is about 3,000 people. The family can bring its own food or pay $5 for food provided at the *Casa Materna*. The family can clean the linens used by the patient there or pay $5 to have them cleaned after the delivery.

**Other Services and Programs at the *Casa Materna***

In addition to delivery care, *Casa Materna* staff provide other services, including prenatal care and facilitation of various education and support groups (e.g., for pregnant women, lactating mothers, adolescent groups). In addition, the staff members provide home visits, meet with local women’s group in the communities (Care Groups and local women’s groups), provide training for community health committees and the micro-regional committees responsible for the *Casas Maternas*, and meet with community assemblies. Discussions are now underway in Guatemala on a broader scale for the MOH to implement this model in hard-to-reach areas inhabited by indigenous populations.

The *Casa Materna* program is linked with a strong outreach delivery system using volunteers to reach every household every 2 weeks to give educational messages and to register vital events. This outreach program follows the census-based, impact-oriented (CBIO) and Care Group methodologies of identifying all homes, visiting all homes on a regular basis with early identification of pregnant women, promotion of healthy behaviors and appropriate health facility utilization (including prenatal care and childbirth at facilities), and registration of vital events.^21^^,^^22^

**Management, Sustainability, and Finances**

The budget of a *Casa Materna* is approximately 20,000 Guatemalan Quetzales (GTQ) per month (about US$2,600, with 7.7 GTQ currently equivalent to US$1). At present, 1 of the 4 *Casas Maternas* has an auxiliary nurse whose salary is paid by the MOH. One of the municipalities is beginning to pay the support workers who work at 1 of the *Casas Maternas*. There are indications that the MOH and the municipalities will gradually support the salaries of the staff at each *Casa Materna*.

The cost of the entire program (including the *Casas Maternas* and the outreach program) is $5.60 per capita per year for the entire project area.

Curamericas recognizes traditional birth attendants as essential partners for improving maternal and newborn care.

A key component of the current program is the *Casa Materna*, which translated literally from Spanish means maternal house. The *Casas Maternas* developed by Curamericas/Guatemala are a response to the lack of readily available around-the-clock delivery care. In the 3 municipalities of the Curamericas program, there was only 1 facility that attended deliveries prior to the operation of the *Casas Maternas*, and it was open only on Mondays through Fridays during the day. This facility was operated by the government and was difficult to access because of the terrain, lack of transportation, and costs involved. Furthermore, staff members were frequently not present and spoke only Spanish, and *comadronas* were not allowed to assist with the delivery.

Casas Maternas, or birthing facilities, were established to provide readily accesible, around-the-clock delivery care to women living in remote areas of Guatemala.

To examine whether the *Casas Maternas* have contributed to increasing health facility deliveries, we conducted a mixed-methods study in San Sebastian Coatán, a municipality with 32 communities, which was part of the 2011 Phase 1 activities. The specific objectives of the study were to examine the degree to which use of *Casas Maternas* has been equitable in terms of family income, educational level of the mother, and distance from the *Casa Materna,* as well as the factors that influenced use of the *Casas Maternas* by women in the communities.

## METHODS

### Study Setting

The study was conducted in the municipality of San Sebastian Coatán, which has 32 scattered mountainous communities, most of which are located at elevations of 2,000–3,800 meters. These communities belong to the *Chuj* ethnic group, which has strong ancestral customs, and they are relatively homogeneous culturally and socioeconomically. Most women speak only the local Mayan dialect (*Chuj*) and only a small proportion speak Spanish.

Until the last decade, most of the communities were inaccessible to vehicles and could be reached only on foot. Still, many of the communities remain accessible only by foot or motorcycle. Levels of education and socioeconomic status are quite low. The area is commonly referred to as the “triangle of death” because of its high mortality rates and because of the oppression it suffered during the Guatemalan civil war from 1960–1996.

There are 2 *Casas Maternas* operating in the study area: one in the small town of Calhuitz, which began operation in 2009, and the other in the town of Santo Domingo, which opened in early 2013. There are 9 and 12 partner communities for the *Casas Maternas* in Calhuitz and Santo Domingo, respectively. The other 11 communities in the study area are non-partner communities. Partner communities were self-selected: (1) they are in close geographic proximity to the *Casa Materna*, (2) the community leaders took on the responsibility of participating in a management committee for the *Casa Materna*, and (3) the community contributed to the construction of the *Casa Materna.* As shown in Figure 1, most but not all, communities surrounding these 2 *Casas Maternas* are partner communities. *Casa Materna* services were available to anyone regardless of community of residence.

### Study Design

A mixed-methods design was chosen to document the extent to which the project’s *Casas Maternas* were used by the surrounding population and to understand how women addressed the complex issues surrounding the decision of where to give birth. Study participants were all of the women who had given birth in the study area during the 12-month period between April 1, 2013, and March 31, 2014, the first full year in which both *Casas Maternas* in the study area were operating, as recorded in the vital events register maintained by the program. The study area consisted of 32 communities of the municipality of San Sebastian Coatán.

Three types of data were collected from the study participants: (1) household survey data from all mothers eligible to be included in the study, (2) in-depth interviews with selected mothers, and (3) focus group discussions with selected key informants, including Micro-Regional Committee members, *comadronas*, and *Casa Materna* staff. The household survey provided the primary data for the socioeconomic status of the household and distance from the *Casa Materna*. These data were complemented by in-depth interviews with individuals and focus group discussions that provided additional insights into what factors were considered in the decision-making process related to birth location, as well as information about the delivery experience and perspectives on the *Casas Maternas*.

### Data Collection

Quantitative data were collected in September 2014 by a survey team of 12 trained women professionals (teachers and health educators) who were fluent both in the local language (*Chuj*) and in Spanish. A structured household questionnaire was administered to all mothers living in the 32 Phase 1 communities in the municipality of San Sebastian Coatán who had given birth during the period from April 1, 2013, until March 31, 2014. The questionnaire included questions about sociodemographic characteristics, including ownership of 22 household assets, as well as information on the location of maternal and newborn health services received. The survey questionnaire was written in Spanish, but the interviews were conducted in *Chuj*.

For the qualitative data collection, key informant interviews and focus group discussions were carried out in 6 purposively selected communities: 2 from non-partner communities (Yoxacla and Chenen) and 4 from partner communities (Calhuitz and Santo Domingo, each of which had a *Casa Materna* in operation, as well as Ulna and Lolbatzan, which did not have a *Casa Materna*). In 5 of the 6 communities, 4 women were purposively selected and interviewed; 2 of the women had used a *Casa Materna* and 2 had not. In the sixth community (Yoxacla), there were no women who had used the *Casa Materna*, so only 2 women were interviewed, both of whom delivered at home. The 22 in-depth interviews were performed by 3 teams of 2 interviewers in the respondents’ homes. The interview covered 3 main topics: (1) how the decision about birth place for the most recent birth was made, (2) the birth experience, and (3) assessment of quality of care and recommendations for improvement of *Casa Materna* services. The inter&1hyphen-qj;views were recorded. Prior to beginning the interview or survey, the interviewer obtained informed consent verified by either a signature or a thumbprint.

Six focus group discussions were conducted with 42 key informants: 2 focus groups were conducted with members of Micro-Regional Committees, 2 with groups of *comadronas*, and 2 with staff of the *Casas Maternas* in the study area. The focus groups were conducted in *Chuj* by 3 teams of 2 trained bilingual women at the Calhuitz and Santo Domingo *Casas Maternas*.

### Data Management

Two field supervisors as well as the data entry supervisor reviewed each completed survey questionnaire to ensure that all questions were clearly and appropriately completed. To de-identify each interview, each questionnaire was labeled with a unique code number. The data were entered into Epi Info version 7.1 by a team of 4 data entry staff trained in Epi Info data entry, quality control, and confidentiality assurance.

For the qualitative data management, one field supervisor as well as the transcription supervisor reviewed each recording and transcription to ensure the quality of both the translation and the transcription. Each recorded session and its corresponding transcription were labeled with a unique code number for quality control and confidentiality.

### Data Analysis

The survey data were analyzed using Epi Info 7.1 and Stata 13. Quantitative data were analyzed using descriptive statistics. Principal components analysis (PCA) was used to create an asset score for each household using 22 household assets according to standard techniques.^11^^,^^12^ For the equity analyses, frequency distributions of the number of years of education of the mother and the distance of the mother’s community from the nearest *Casa Materna* were used to establish terciles. The distribution of the family’s PCA asset scores was used to develop quintiles. For education, the terciles were defined as follows: 0 years of education, 1–3 years, and 4 or more years (30%, 33%, and 37% of the respondents, respectively). Distance terciles were also defined: 0 to <4 km, 4 to <8 km, and 8–15 km (34%, 33%, and 33% of the respondents, respectively). The percentage falling in each wealth quintile ranged from 19% in the first quintile to 21% in the third.

Equity of health facility utilization was assessed by calculating the number of women in each equity tercile (or quintile) who gave birth in a facility divided by number of women in that group who had given birth during the year of the study.

Although all women giving birth between April 1, 2013, and March 31, 2014, in the study population were included in the 2014 household survey (i.e., no sampling was carried out), conservative estimates of confidence intervals were calculated using WIN-PEPI and Epi Info 7.1.

The qualitative data were manually reviewed and inductively grouped and coded into categories as shown in Table 1. This process is referred to as descriptive content analysis, which involves identifying themes among the responses and locating the specific responses with these themes. The research team then checked the coding and interpretation collaboratively and found them to be consistent.

**TABLE 1:** Framework for Qualitative Data Analysis

Theme	Description
Decision about birth place	Comments about how the location of birth was selected
	**Sub-codes:** Influence of others on delivery location Cultural traditions Previous birth experiences Perception of distance to the facility and availability of transportation Influence of costs on birth location
Birth experience	Comments related to the actual experience of giving birth
	**Sub-codes:** Cultural traditions Opinions about the care received at home or at a facility
Recommendations	Suggestions for improving labor and delivery services at the *Casa Materna*

The quantitative and qualitative data were integrated at the interpretation stage and triangulated for congruence and complementarity.

### Ethical Clearance

The study protocol was approved by the National Committee for Ethics in Health of the Ministry of Public Health and Social Welfare of Guatemala. All research participants provided written or verbal informed consent.

## RESULTS

### Quantitative Results

#### Demographic Characteristics of Study Respondents

Of the 321 women identified from the vital events system as giving birth during the study period, 46 (14.3%) had moved out of the community, could not be located, or refused to be interviewed. A total of 275 women were interviewed. The participation rate of these women from the partner communities (86%) was virtually identical as that from women in the non-partner communities (85%).

The demographic characteristics of the survey respondents are presented in Table 2. In brief, nearly all (99%) respondents were of *Chuj* ethnicity, with a mean age of 25 years and an average of 3 children. The mean level of education was 2.7 years. Only 15% of their families owned a vehicle or motorcycle; most depended on local private minibus and bus services.

**TABLE 2:** Demographic Characteristics of Survey Respondents (N = 275), by Type of Community

Demographic Characteristic	All Respondents (N = 275)	Partner Communities (n = 189)	Non-Partner Communities (n = 86)	*P* Value^a^
Age of mother, mean, years	25.2	25.3	25.0	.93
No. of persons in household, mean	6.8	6.9	6.6	.76
No. of children in household, mean	3.2	3.4	3.0	.12
Living with spouse/partner, %	87.3	89.9	81.4	**.049**
Speak *Chuj*, %	98.9	98.9	98.8	.94
Speak Spanish, %	24.7	27.0	19.8	.20
Principally housewives, %	95.3	95.2	95.3	.71
Family received remittances in past 3 months, %	20.4	20.6	19.8	.87
Reported food insecurity in past 6 months, %	21.8	21.7	22.1	.94
Family owns a vehicle or motorcycle, %	14.5	14.3	15.1	.72
No. of years of education of mother, mean	2.7	2.8	2.6	.66
PCA asset score of household, mean	0.97	0.96	1.00	.55
Distance of mother's community from nearest *Casa Materna*, mean, km	5.4	4.1	8.3	**<.01**

Abbreviation: PCA, principal components analysis.

a Comparing partner communities with non-partner communities. Values shown in boldface are statistically significant at *P*<.05.

The characteristics of the population in the partner communities were similar to those in the non-partner communities except that non-partner communities were considerably further away from a *Casa Materna* (Table 2). The mean distance of a respondent’s community to the nearest *Casa Materna* was 4 km for those living in partner communities compared with 8 km for those living in non-partner communities (*P*<.01). This was expected as the *Casas Maternas* are strategically located to be in proximity to their partner communities. In the non-partner communities, no women lived in the closest tercile (0 to <4 km from the nearest *Casa Materna*), while 68% of the women lived in the farthest tercile (8–15 km).

#### Utilization of Health Facilities for Deliveries

Among the 189 women in our study who resided in partner communities, 69.8% reported that they delivered their child in a health facility (54.4% in a *Casa Materna*) during the period from April 2013 through March 2014 (Table 3). In contrast, only 30.2% of the study participants from non-partner communities reported delivering in a health facility (17.4% in a *Casa Materna*) during the same time period.

70% of women from partner communities reported delivering in a facility compared with 30% of women from non-partner communities.

**TABLE 3:** Location of Deliveries During the Study Period (April 1, 2013–March 30, 2014) in the San Sebastian Coatán Municipality, by Type of Community

Location of Delivery	All Respondents (N = 275)	Partner Communities (n = 189)	Non-Partner Communities (n = 86)
**Non-health facility deliveries**	**117 (42.5)**	**57 (28.1)**	**60 (69.8)**
Home of interviewee or of another person	116 (42.1)	56 (29.6)	60 (69.8)
En route in ambulance	1 (0.4)	1 (0.5)	0 (0.0)
**Health facility deliveries**	**158 (57.5)**	**132 (69.8)**	**26 (30.2)**
Hospital	18 (6.5)	13 (6.9)	5 (5.8)
MPHSW health center	20 (7.3)	14 (7.4)	6 (7.0)
Private clinic	2 (0.7)	2 (1.1)	0 (0.0)
* Casa Materna* Calhuitz	74 (26.9)	59 (31.1)	15 (17.4)
* Casa Materna* Santo Domingo	44 (16.1)	44 (23.3)	0 (0.0)

All data shown as No. (%).

Abbreviation: MPHSW, Ministry of Public Health and Social Welfare.

We do not have comparable baseline data from 2011 (when the project began), but we do have data from 2 household knowledge, practice, and coverage (KPC) surveys carried out in January 2012. (Although the Calhuitz *Casa Materna* had been in operation since 2009, utilization of the Calhuitz *Casa Materna* during its first 2 years of operation was quite low, picking up substantially in 2011; thus, one can assume 2011 as the baseline for the Calhuitz location as well.) The 2012 KPC data indicate that only 16% of women with a child younger than 2 years of age in the Phase 1 project area (comprising 89 communities in 3 municipalities) reported that their most recent birth occurred in a facility; in the Phase 2 project area (comprising 94 communities in 3 municipalities), the percentage was only 7%. In addition, of the 50 women living in partner communities who participated in the January 2012 baseline KPC survey, 32.0% reported a facility delivery at the time of their most recent delivery. Although none of these data are directly comparable, they do suggest that the percentage of facility births taking place in partner communities increased substantially in a short period of time, from January 2012 to the study period (April 2013 through March 2014).

#### Equity of Health Facility Utilization

**FIGURE 2.**
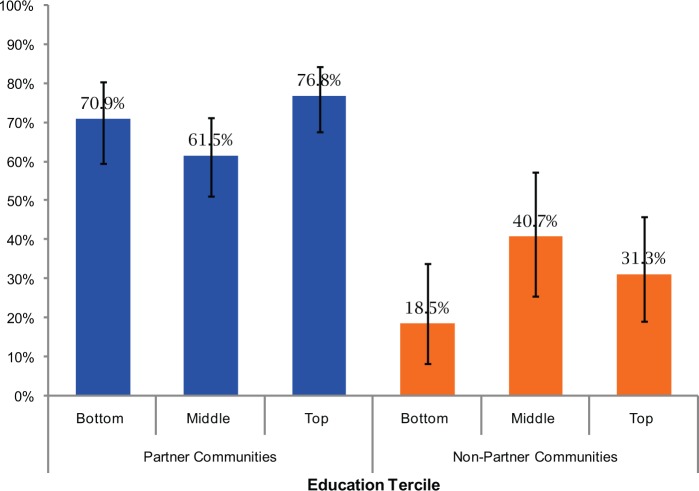
Percentage of Health Facility Deliveries by Women’s Education Tercile^a^ and by Type of Community 95% confidence intervals shown. ^a^ Bottom education tercile, no education; middle tercile, 1–3 years of education; top tercile, 4+ years.

Figure 2 shows the percentage of women delivering in a facility by level of education of the mother for partner and non-partner communities. In neither the partner communities nor the non-partner communities was there a statistically significant difference in the percentage of health facility births by educational level for women, suggesting that those with the lowest level of education were as likely to obtain a facility birth as those with higher levels of education. However, in the non-partner communities, there was a suggestion of increasing health facility utilization among those with more education, increasing, for example, from 18.5% among the bottom education tercile to 40.7% in the middle education tercile.

**FIGURE 3.**
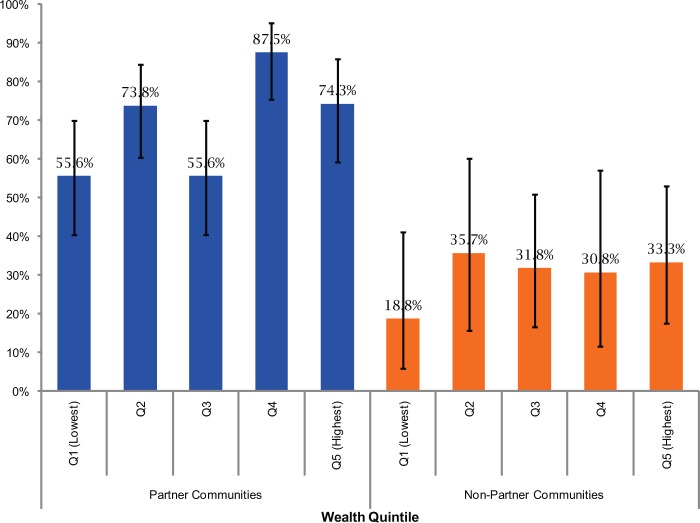
Percentage of Health Facility Deliveries by Women’s Household Wealth Quintile and by Type of Community 95% confidence intervals shown.

Similar patterns were observed by wealth quintile (Figure 3), although in the partner communities there was a suggestion of a modest effect of increased utilization among women in only one of the 4 wealthier quintiles, with the difference between the lowest and the fourth quintile reaching statistical significance (*P*<.01). In the non-partner communities, the lowest wealth quintile had lower utilization of health facilities (18.8%) than the other wealth quintiles (range, 30.8% to 35.7%), but the difference was not statistically significant.

**FIGURE 4.**
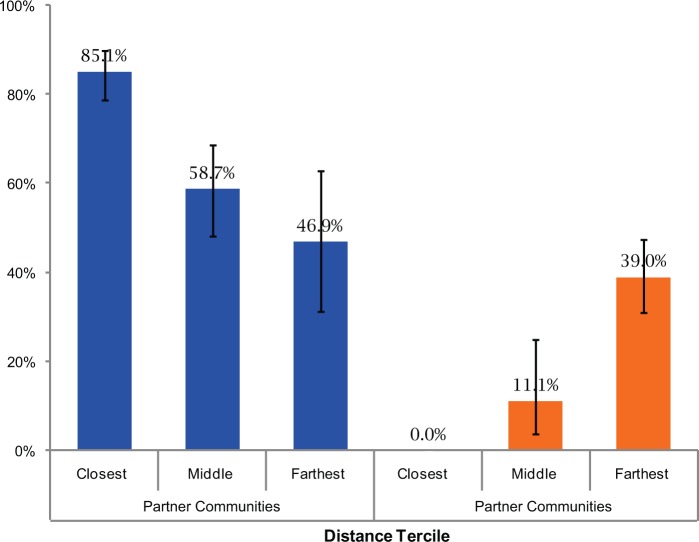
Percentage of Health Facility Deliveries by Distance of Women’s Household to the Nearest *Casa Materna* and by Type of Community 95% confidence intervals shown. Closest tercile is <4 km from the *Casa Materna*; middle tercile is 4 to <8 km; farthest tercile is ≥8 km away.

The effect of distance from the *Casa Materna* on birth location present a more complex picture (Figure 4). For all the partner communities, the greater the distance, the lower the facility delivery coverage rate. Among the non-partner communities, none of the women giving birth in a facility lived within 3 km of a *Casa Materna* (i.e., the closest tercile). For the intermediate and most distant tercile groups, delivery rates were actually higher among the most distant group (39.0%) than among the intermediate group (11.1%). Almost one-quarter of the births in the most distant tercile of the non-partner communities occurred at the Calhuitz *Casa Materna.*

### Qualitative Results

For qualitative data from in-depth interviews and focus groups, first-order descriptive themes and second- and third-order analytic themes are summarized in Table 4. The findings for the main topics are described in the following sections.

**TABLE 4:** Thematic Analysis of Factors Affecting Decision About Birth Place

First-Order Themes	Second-Order Themes	Third-Order Themes
**Barrier:** Too many people involved in the decision-making process	Influence of others on delivery location	Decision about birth place
**Facilitator:** The role of *comadronas*		
**Facilitator/barrier:** The role of husbands		
**Barrier:** Tradition supports giving birth at home	Cultural traditions	
**Facilitator:** The role of the *comadrona*		
**Facilitator/barrier:** Effects of previous birth experiences on subsequent delivery location	Previous birth experience	
**Barrier:** *Casa Materna* perceived as being located far away by some	Perception of distance	
**Facilitator:** *Casa Materna* perceived as being nearby by others		
**Barrier:** Perceived high cost of facility birth compared with home birth	Cost of childbirth	
**Facilitator:** Perceived low cost of *Casa Materna* for facility birth compared with private facilities		
**Facilitator:** *Comadrona* is part of the team during delivery process	Cultural traditions	Assessment of birth experience
**Facilitator:** Woman is attended to in her own language and with respect for long-standing traditions		
**Barrier:** Home perceived as safe place to give birth	Perceived quality of care	
**Facilitator:** Home perceived as an unsafe place to give birth.		
**Facilitator:** *Casa Materna* perceived as providing high-quality care		
**Facilitator:** To have more equipment	Suggestion	Recommendations for improving care at the *Casa Materna*

#### Decision About Birth Place

Many people were identified as being involved in the process of decision making about the birthing place, and the woman herself was generally not the final decision maker. The *comadrona* and the husband were identified as playing key decision-making roles. The *comadrona* was found to be one of the best supporters of the *Casa Materna* and a strong motivator for women to have their deliveries there. A woman living in a partner community explained:

When I knew I was pregnant I told the comadrona, and she advised me to go to the Casa Materna and the entire family accepted my decision.

Comadronas and husbands were identified as playing key decision-making roles about where a woman would deliver.

A *comadrona* living in a partner community described the rationale she gave to women to deliver at the *Casa Materna*:

Before I accepted the management of the pregnancy, I advised the woman and her family that she must go to the Casa Materna because there could be complications in childbirth.

In some settings, the husband acted as a facilitator by supporting his wife in her decision to use the *Casa Materna*, while in others, the husband prohibited a facility delivery due to cultural traditions such as *machismo* (a cultural tradition that embraces the subjugation of women by men that is expressed in attitudes, behaviors, and decisions). In still other settings, the husband played a more neutral role and placed the decision making in someone else’s hands, such as an elder female family member. A woman from a non-partner community explained that the final decision was in her husband’s hands:

My husband made the decision that I give birth at home and said that I should not go elsewhere.

Other factors such as cultural traditions and previous experience play an important role in discouraging use of the *Casa Materna.* For example:

*I decided to give birth at home because I have 3 children and have experience. It is the custom to give birth at home.* –Woman living in a non-partner community

Geographic distance to the *Casa Materna* was also found to be an influential factor affecting delivery location. The perceived far distance, as well as the lack of or high cost of transportation, influenced the decision for some women. Some women reported that the *Casa Materna* was too far to travel to during labor, and travel at night or during the rainy season was also considered particularly difficult. Perception of distance in some cases was more important than actual distance: some perceived that the *Casa Materna* was close to their community even when the community was more than 8 km from the *Casa Materna*.

*I have no possibility to go to the Casa Materna because I have no money; it is far and transportation is expensive.* –Woman living in a partner community

*I went to the Casa Materna since not much money is spent because it is near my community.* –Woman living in a non-partner community

#### Assessment of the Childbirth Experience

Key informants reported that when a woman goes into labor, the *comadrona* is contacted and then comes to the home. She then either attends the woman’s birth at home or accompanies her to the *Casa Materna* with her husband and family members, depending on the family’s decision. In the partner communities, the *comadrona* is considered as part of the team but is not formally a member of the staff of the *Casa Materna*.

*When the labor pains started I told my husband and he called my comadrona. My husband looked for transportation and we went to the Casa Materna.* –Woman living in a partner community

*The Casa Materna cares for us well since the staff understand our culture, which is not the case in the government hospital.* –Comadrona living in a partner community

Women who perceived that the *Casas Maternas* provide high-quality care reported feeling more comfortable giving birth at a *Casa Materna*. Community leaders and *comadronas* also reported feeling comfortable working with *Casa Materna* staff members because of the quality of care that they provide. Staff of the *Casa Materna* reported that the participation of the *comadronas* during the delivery process was helpful and contributed to good outcomes.

*I chose the Casa Materna to have a safe delivery and avoid any complications during delivery.* –Woman living in a non-partner community

*Our communities have benefited from the Casa Materna because now our women have a clean, safe place to give birth to our children.* –Leader in a partner community

*The Casa Materna has the necessary medicines to attend births as well as good attention from the nurses, and they allow me to be with her [my patient] during the labor.* –Comadrona in a partner community

*In the past our children were born into garbage, but now they are born into cleanliness.* –Member of the Santo Domingo Micro-Regional Committee

#### Recommendations for Improvement

Respondents from both in-depth interviews and focus groups agreed that the *Casas Maternas* provide good-quality care. However, some of the respondents did recommend that the *Casas Maternas* should provide sonography.

## DISCUSSION

The purpose of the present study was to examine whether *Casas Maternas* have contributed to increasing health facility deliveries in an equitable manner in 32 communities of the municipality of San Sebastian Coatán and what factors have influenced use of the *Casas Maternas* by women in the communities. Our findings clearly indicate that there is a relatively high rate of utilization of the *Casas Maternas.* By 2014, 54% of women living in the Calhuitz and Santo Domingo partner communities were giving birth at the respective *Casas Maternas.*

These findings are particularly impressive in light of the low percentage of births (21%) taking place at facilities in the overwhelmingly indigenous department of Huehuetenango and the low percentage of facility births (29%) among indigenous women in the country as a whole.^6^ *Casas Maternas* are clearly increasing the percentage of facility births occurring among indigenous women in a rural isolated area in the Western highlands of the department of Huehuetenango and therefore are beginning to contribute to reducing the national inequities that exist in this regard.

Casas Maternas have achieved local equity in provision of facility deliveries in terms of wealth and education of mothers.

The data also show that within this context of poverty and limited education, local equity in the provision of health facility deliveries with respect to relative wealth and education was achieved. Although the study population is relatively homogenous in terms of education (but less so in terms of income), our findings indicate that utilization of health facilities for delivery is similar across education terciles and wealth quintiles in both partner and non-partner communities. However, *Casa Materna* utilization is more equitable (in terms of education and wealth) than utilization of non-*Casa Materna* health facilities (data not shown).

But the data also clearly demonstrate that in this extreme geography, distance matters. Living in a partner community within 3 km of a *Casa Materna* greatly increased the likelihood that a woman would deliver in the *Casa Materna* and benefit from a clean and safe health facility delivery. The qualitative data support this finding, as women who did not use the *Casa Materna* often cited the perceived or real distance and cost of transportation as barriers. Our findings echo that of other studies that have highlighted the importance of close-to-home birthing facilities to expand facility deliveries.^13^^,^^14^

Distance matters: women in partner communities who lived close to a Casa Materna had higher facility delivery rates than women living farther away.

Although our study did not explore quality of care directly, it is important to point out that there were no maternal deaths among the 189 participants who gave birth during the study period (April 1, 2013, through March 31, 2014) who were living in the partner communities that support the 2 *Casas Maternas*. In contrast, there were 3 maternal deaths among the 86 study participants in the non-partner communities (*P* = .03). Furthermore, there were no maternal deaths among the 206 deliveries that specifically took place in the 2 *Casas Maternas* during the study period. These findings add credence to the *Casa Materna* model described in this paper. Forthcoming publications will review specific issues related to the quality of care and overall impact of the 4 *Casas Maternas* in the project area following their introduction, including more recent data through 2015.

The literature on *Casas Maternas* in the Americas is limited, but there are 2 recent examples in which similar approaches have been tried unsuccessfully.^15^^,^^16^ In both, community engagement and community ownership were absent, suggesting that these factors—which were critical for establishing the operation of the *Casas Maternas* (construction and management of the facility)—are particularly important for explaining the success of the *Casas Maternas* in the Curamericas program area of Huehuetenango. In addition, anecdotal evidence provided by project staff suggests that the outreach component of the Curamericas program (visiting all homes for promotion of healthy behaviors and appropriate utilization of health facilities) has encouraged mothers to deliver in facilities. Other contributory success factors include the close location of services to families compared with those provided at government facilities and the community’s perception of high-quality services provided in the *Casas Maternas*—that women are treated with respect, that the care is culturally appropriate, and that the care is of good medical quality. Finally, the *comadronas* appear to have played an important role in influencing women to give birth in a *Casa Materna,* with the qualitative data from our study demonstrating that the strong encouragement of health facility deliveries by the *comadronas* was decisive for many women.

Community engagment and ownership are important success factors to the Casas Maternas program.

*Comadronas* seem to be enthusiastic in supporting the use of the *Casa Materna* for 4 reasons: (1) they are not losing any income by promoting use of the *Casa Materna*, (2) they continue to play an important role in providing support to the mother and her family and in participating in the delivery itself, (3) they do not suffer the risk of being blamed for any complication that might arise, and (4) they are beginning to realize that delivery in a *Casa Materna* is in the best interest of the mother and her child.

### Opportunities for Incorporating *Casa Materna* Principles Into the MOH System

Although it is not easy to address all of the many and complex factors contributing to disparities in maternal mortality in Guatemala within the government system, the MOH is beginning to take steps to incorporate some of the principles of *Casas Maternas* within the department of Huehuetango by converting peripheral health posts (each serving 3,000–5,000 people) in 2 municipalities of the Western highlands of the department into *Casas Maternas.* These facilities, staffed by auxiliary nurses who are from the area and speak the local language, will now be open continuously (24 hours a day, 7 days a week), and *comadronas* will be welcome to accompany their patients for delivery there. The MOH is now setting up for the first time community committees to provide oversight into the functioning of these health posts. Finally, these health posts are intended to become a type of local “emergency room,” where patients with acute illnesses can be seen and treated or referred. Access to referral through telephone communication and prearranged transport is also being developed. If successful, this approach could be scaled-up more broadly at the national level.

The government of Guatemala is beginning to incorporate Casa Materna principles into the ministry system.

### Relevance of the Findings for Guatemala and Beyond

The issues faced by the women of the project area are not uncommon throughout Latin American countries with indigenous populations that have been marginalized over the centuries following the Spanish conquest, particularly those living in more isolated mountainous communities. Moreover, many of these same issues are faced by other poor women in low- and middle-income countries around the world, especially those living in isolated rural locations.

Globally, one-third of births still occur in the home. In sub-Saharan Africa, South Asia, and in the least-developed countries, the percentages are 46%, 45%, and 44%, respectively.^17^ In spite of major pushes by governments to promote facility-based deliveries, progress has been slow. One of the reasons for this has been the lack of readily accessible, locally accountable, and “community-friendly” facilities staffed by local people where local traditions and norms are respected.

A recent review synthesizing the qualitative evidence regarding facilitating factors and barriers to facility-based deliveries in low- and middle-income countries concluded that women and their families in many settings have come to believe that “childbirth has become medicalized and dehumanized” and that families avoid facilities for childbirth because of a fear of undesirable procedures as well as fear of disrespectful and abusive care.^18^ Evidence of mistreatment of women during childbirth in health facilities is abundant according to a recent systematic review.^19^ In 2014, the World Health Organization released a statement on the prevention and elimination of disrespect and abuse during facility-based childbirth that has been endorsed by leading organizations around the world involved in women’s health, recognizing the right of every woman to dignified, respectful health care.^20^ The statement recognized that those at greatest risk of disrespectful treatment and abuse are adolescents, unmarried women, women from ethnic minorities, migrant women, and women living with HIV.

One recent review^21^ of a similar approach to the *Casas Maternas* in rural Nepal that uses skilled birth attendants in birthing centers identified 2 major drawbacks of the approach from the standpoint of the providers: the skilled birth attendants worked alone and access to referrals was lacking. The *Casa Materna* approach overcomes these 2 drawbacks since a team of providers is always available along with prompt referral.

The approach developed by Curamericas in the rural highlands of Guatemala to expanding access to respectful, culturally appropriate facility-based childbirth is now gaining attention in other similar areas of the country, and plans are underway to develop *Casas Maternas* there. The approach has relevance not only for Guatemala and Latin America but also for areas of sub-Saharan Africa and South Asia where home deliveries still predominate. The *Casa Materna* model developed by Curamericas in Guatemala offers an important example of how communities can engage with health systems to establish “community-friendly” spaces for delivery by more highly trained personnel than traditional birth attendants while at the same time honoring the role of traditional birth attendants.

The Casa Materna model offers an example of how to engage communities to establish community-friendly spaces for high-quality facility deliveries while honoring the role of traditional birth attendants.

Whether or not the increased availability and utilization of “community-friendly” birthing centers reduces maternal and neonatal mortality in the Curamericas project area, in Guatemala or elsewhere, remains an open question. The answer will depend on the capacity of the *Casas Maternas* to prevent infection, manage complications, and facilitate referral to the next level of care—issues that will be addressed further in subsequent publications along with questions related to costs and sustainability.

### Study Limitations

Our study has several limitations. First, it would have benefited from direcly comparable baseline data on the characteristics of women using health facilities prior to introduction of the *Casas Maternas* to better assess whether improvements in equity as well as coverage had occurred. Second, because the number of surveyed respondents was limited (N = 275), the ability to detect statistically significant differences among variables influencing *Casa Materna* utilization was limited. Third, in the translation of interview questions and answers from Spanish to *Chuj,* back to Spanish, and ultimately to English, some important meanings could have been lost, despite having bilingual Spanish/*Chuj* and Spanish/English staff performing the translations. Finally, stronger evidence on the quality of maternity and newborn care provided in the *Casas Maternas* and health outcomes of patients receiving care there (including maternal and perinatal mortality) would have enabled us to make a stronger case for the applicability of the *Casa Materna* model on a broader scale. Ongoing services provided at *Casas Maternas* are being monitored, and future reports will address in greater detail the quality of care provided there. As indicated elsewhere, the number of *Casas Maternas* in the project area is growing and the Ministry of Health is beginning to convert some of its health posts in the project area into quasi-*Casas Maternas.* Plans are also underway to convert health posts in another department (San Marcos) into quasi-*Casas Maternas*, and several other NGOs are in the process of establishing *Casas Maternas* based on the Curamericas model. Thus, these experiences will help to shape the evidence regarding the advisability of scaling-up the *Casa Materna* approach more broadly.

## CONCLUSION

Working with communities to establish *Casas Maternas* that provide high-quality, culturally appropriate, and readily accessible maternity care in an isolated mountainous area of Guatemala, where most births are still attended at home by traditional birth attendants (*comadronas*), provides a promising approach to increasing facility-based deliveries at low cost. Uptake of these services was equitable in terms of maternal education and family income but was not able to fully overcome geographic barriers for those who live at greater distances. *Casas Maternas* also provide opportunities for *comadronas* to continue in their traditional role of supporting mothers at the time of childbirth while also serving as champions of facility delivery. Linking strong, frequent outreach to all households by volunteers through the CBIO+ Care Group approach^22^^,^^23^ with *Casas Maternas* also warrants consideration for broader application in Guatemala and beyond.

## Notes

**Peer Reviewed**
